# DeepDixon synthetic CT for [^18F]FET^ PET/MRI attenuation correction of post-surgery glioma patients with metal implants

**DOI:** 10.3389/fnins.2023.1142383

**Published:** 2023-04-06

**Authors:** Claes Nøhr Ladefoged, Flemming Littrup Andersen, Thomas Lund Andersen, Lasse Anderberg, Christian Engkebølle, Karine Madsen, Liselotte Højgaard, Otto Mølby Henriksen, Ian Law

**Affiliations:** Department of Clinical Physiology and Nuclear Medicine, Rigshospitalet, University of Copenhagen, Copenhagen, Denmark

**Keywords:** AI, attenuation correction, deep learning, DeepDixon, glioma, post-surgery, PET/MRI

## Abstract

**Purpose:**

Conventional magnetic resonance imaging (MRI) can for glioma assessment be supplemented by positron emission tomography (PET) imaging with radiolabeled amino acids such as O-(2-[^18^F]fluoroethyl)-L-tyrosine ([^18^F]FET), which provides additional information on metabolic properties. In neuro-oncology, patients often undergo brain and skull altering treatment, which is known to challenge MRI-based attenuation correction (MR-AC) methods and thereby impact the simplified semi-quantitative measures such as tumor-to-brain ratio (TBR) used in clinical routine. The aim of the present study was to examine the applicability of our deep learning method, DeepDixon, for MR-AC in [^18^F]FET PET/MRI scans of a post-surgery glioma cohort with metal implants.

**Methods:**

The MR-AC maps were assessed for all 194 included post-surgery glioma patients (318 studies). The subgroup of 147 patients (222 studies, 200 MBq [^18^F]FET PET/MRI) with tracer uptake above 1 ml were subsequently reconstructed with DeepDixon, vendor-default atlas-based method, and a low-dose computed tomography (CT) used as reference. The biological tumor volume (BTV) was delineated on each patient by isocontouring tracer uptake above a TBR threshold of 1.6. We evaluated the MR-AC methods using the recommended clinical metrics BTV and mean and maximum TBR on a patient-by-patient basis against the reference with CT-AC.

**Results:**

Ninety-seven percent of the studies (310/318) did not have any major artifacts using DeepDixon, which resulted in a Dice coefficient of 0.89/0.83 for tissue/bone, respectively, compared to 0.84/0.57 when using atlas. The average difference between DeepDixon and CT-AC was within 0.2% across all clinical metrics, and no statistically significant difference was found. When using DeepDixon, only 3 out of 222 studies (1%) exceeded our acceptance criteria compared to 72 of the 222 studies (32%) with the atlas method.

**Conclusion:**

We evaluated the performance of a state-of-the-art MR-AC method on the largest post-surgical glioma patient cohort to date. We found that DeepDixon could overcome most of the issues arising from irregular anatomy and metal artifacts present in the cohort resulting in clinical metrics within acceptable limits of the reference CT-AC in almost all cases. This is a significant improvement over the vendor-provided atlas method and of particular importance in response assessment.

## 1. Introduction

Conventional magnetic resonance imaging (MRI) is the imaging modality of choice in neuro-oncology, both in clinical practice and in clinical trials (Holzgreve et al., 2021), and is used primarily for qualitative subjective interpretation with simplified measures such as size, number of lesions, and contrast enhancement patterns (Mullins et al., 2005; Galldiks et al., 2015a; Smits, 2021). When MRI is challenged in glioma assessment it may be supplemented by positron emission tomography (PET) imaging with radiolabeled amino acids, such as O-(2-[^18^F]fluoroethyl)-L-tyrosine ([^18^F]FET), that provide additional information on metabolic properties by visualizing the L-amino acid transporter (LAT) expression. The recommended clinical use of amino acid PET according to recent guidelines includes differential diagnosis in the primary evaluation of brain lesions, tumor grading, biopsy optimization and the differentiation of tumor relapse from treatment-related changes (Albert et al., 2016; Law et al., 2019). Pathological amino acid accumulation is estimated using simplified semi-quantitative measures such as tumor-to-brain ratio (TBR) on static [^18^F]FET-PET images, and by evaluating the tracer time-activity curve (TAC) extracted from dynamic images for 40–50 min following tracer administration (Suchorska et al., 2016; Law et al., 2019). Other indications such as response assessment and treatment planning of radiation and surgical intervention rely on the ability of amino acid PET to identify the extent of infiltrating glioma expressed as the biological tumor volume (BTV) (Law et al., 2019). The BTV is prognostic for overall survival in post-resection glioblastoma multiforme in multivariate analysis (Poulsen et al., 2017).

A prerequisite for diagnostic and prognostic accuracy and response assessment is quantitatively correct [^18^F]FET-PET images, which among others requires accurate attenuation correction (AC) (Vander Borght et al., 2006). In simultaneous PET/MRI, AC is usually performed using an MRI-based method, which poses a challenge since MRI is not related to electron densities contrary to e.g., computed tomography (CT) scans used for AC of PET/CT examinations. Several MR-AC methods exist, however, only a few of these methods are made available by the vendors in clinical brain PET/MRI (Ladefoged et al., 2016; Catana, 2020). For both the Biograph mMR scanner (Siemens Healthineers, Erlangen, Germany) and the Signa PET/MRI scanner (GE Healthcare, Chicago, IL, USA) there are atlas-based methods available, where a probabilistic atlas created from a database of CT images is aligned to MRI (Wollenweber et al., 2013; Paulus et al., 2015; Koesters et al., 2016), and additionally the option of a segmentation-based method, where dedicated short echo time sequences are used to separate voxels corresponding to air, tissue and bone (Keereman et al., 2010; Wiesinger et al., 2016).

While the performance of these methods has been evaluated extensively (Dickson et al., 2014; Ladefoged et al., 2016; Sekine et al., 2016a,b, 2020), the majority of these studies are based on dementia cohorts consisting of patients with normal skull anatomy. In neuro-oncology, patients often undergo brain and skull altering treatment, which might challenge atlas-based methods due to a lack of representative cases in the atlas database. While segmentation-based approaches might be more suited for adapting to abnormal anatomy, these methods are in turn susceptible to metal implant-induced artifacts, which depending on the sequence, can lead to distortions and/or partial or complete signal loss in the MRIs (Hargreaves et al., 2011).

We have previously demonstrated that RESOLUTE (Ladefoged et al., 2015), a segmentation-based method, can reproduce the clinical metrics of CT-AC in neuro-oncology studies, albeit with some outliers caused by local artifacts around titanium alloy mesh implants (Ladefoged et al., 2017). The advances in deep learning have led to numerous methods utilizing convolutional neural networks (CNNs) for MRI-to-CT conversion (Teuho et al., 2020; Torrado-Carvajal, 2020). We proposed such a method for MR-AC of pediatric neuro-oncology patients, which was found to improve the performance over RESOLUTE even in cases with irregular anatomy such as post-operative subcutaneous soft tissue swelling (Ladefoged et al., 2019). Finally, we proposed DeepDixon, a CNN identical to our pediatric version but re-trained and evaluated using a non-surgical cohort consisting of 1,037 adult subjects, resulting in average PET bias below 1% in any region of the brain (Ladefoged et al., 2020).

The aim of the present study was to examine the applicability of DeepDixon for MR-AC in [^18^F]FET PET/MRI scans of a post-surgery glioma cohort with metal implants, which represents the group of patients with the most challenging abnormal anatomy and metal-induced artifacts in clinical brain PET imaging. DeepDixon and the vendor-standard atlas-based method were compared to CT-AC and evaluated using the recommended clinical metrics on a patient-by-patient basis.

## 2. Materials and methods

### 2.1. Patients

The department imaging archive was screened for patients above 18 years with prior surgery for histologically proven glioma classified according to recent guidelines (Louis et al., 2016, 2021) and a simultaneous [^18^F]FET-PET and MRI scan performed using our hybrid PET/MRI system between October 2018 and January 2021. A total of 194 patients (mean age 54 years; range: 18–80 years) with in all 318 PET/MRI scans met the inclusion criteria (Table 1).

**TABLE 1:** Patient characteristics for all studies as well as for a subgroup of patients with BTV above 1 ml which were included in the PET evaluation.

	All included patients	Evaluated patients (BTV > 1 ml)
**Characteristic**	**PET/MRI studies (No. of patients)**	**PET/MRI studies (^#^patients)**
*n*	318 (194)	222 (147)
Male	173 (105)	121 (82)
Female	145 (89)	101 (65)
Anaplastic pilocytic astrocytoma	3 (2)	3 (2)
Anaplastic pleomorphic xanthoastrocytoma	2 (1)	2 (1)
Astrocytoma, WHO grade 3	36 (23)	19 (13)
Glioblastoma, WHO grade 4	229 (138)	171 (115)
Oligodendroglioma, WHO grade 2	11 (5)	8 (3)
Oligodendroglioma, WHO grade 3	21 (16)	14 (9)
Other*	16 (9)	5 (4)

*Other include patients with pleomorphic xanthoastrocytoma, diffuse midline glioma H3 K27M mutant and more. Molecular characteristics are not included as they are not essential to this study.

### 2.2. Acquisition of CT

A reference low-dose CT image (120 kVp, 30 mAs, 74 slices, 0.6 mm^3^×0.6 mm^3^×3 mm^3^ voxels) of the head using a whole-body PET/CT system was used (Biograph TruePoint 40 and Biograph TruePoint 64, Siemens Healthineers, Knoxville, USA). The CT images were acquired on the same day as the PET/MRI examination (*n* = 123), or at a previous examination (*n* = 195) at a median of 112 days before (range 14–1,120 days) with no brain or skull altering surgery in-between.

### 2.3. Acquisition of MRI

The scan protocol included a T1-weighted (T1w) MPRAGE and a Dixon-VIBE sequence (the vendor default for MR-AC) with repetition time (TR) 4.14 ms, echo time 1 (TE1) 1.28 ms, echo time 2 (TE2) 2.51 ms, flip angle 10 degrees, coronal orientation, 39 s acquisition time, voxel size of 1.3 mm^3^×1.3 mm^3^×2.0 mm^3^. The software version was VE11P for all subjects.

### 2.4. Acquisition of [^18^F]FET-PET

Patients were positioned head first with their arms down on the fully integrated PET/MRI system. Data were acquired in list mode over 20–40 min (or 0–40 min for a subset of patients) after injection of 200 MBq [^18^F]FET over a single bed position of 25.8 cm covering the head and neck. For the purpose of this study, the PET data from the PET/MRI acquisition were reconstructed offline (E7tools, Siemens Healthineers, Knoxville, USA) using 3D ordinary poisson-ordered subset expectation maximization (OP-OSEM) with 4 iterations, 21 subsets, zoom 2.5, and a 5 mm Gaussian post-filtering on 344 × 344 matrices (0.8 mm^3^ × 0.8 mm^3^ × 2.0 mm^3^ voxels) in line with the clinical protocol used at our institution. We reconstructed the summed 20-min PET image for all patients (over 20–40 min for the patients imaged over 0–40 min), and in addition for the subset with 0–40 min dynamic imaging, we also reconstructed a dynamic series split into 14 frames (5 min × 1 min, 5 min × 3 min, 4 min × 5 min) similar to Galldiks et al. (2015b). For all images default random, scatter, and dead time correction were applied.

### 2.5. Attenuation correction methods

Three methods for AC were applied to the data: (1) the CT image rigidly aligned to the T1w MPRAGE were used as gold standard AC reference (Andersen et al., 2014), (2) the vendor-provided atlas-based attenuation map that incorporates spatially variant attenuation coefficients of the major bone structure into the Dixon-VIBE MR-AC (Paulus et al., 2015; Koesters et al., 2016), and (3) our deep learning-based method DeepDixon.

DeepDixon is a method to synthesize a brain CT image from only the fat- and water-weighted images of the Dixon-VIBE sequence. The method was developed and validated using more than 1,000 adult subjects primarily referred with suspicion of dementia, where a clinical evaluation using an independent test set showed no relevant differences compared to CT-AC (Ladefoged et al., 2020). The subjects used to develop DeepDixon all had normal anatomy, and thus, the method was not specifically trained to overcome the challenges related to imaging patients with brain surgery, e.g., bone modifying cranio-facial surgical interventions, cranial defects, dysplasias, disfigurements or metal implants besides dental implants. None of the patients included in this present study were part of the dataset used to develop or evaluate DeepDixon.

We did not re-train DeepDixon for the purpose of this study, but directly applied the model to generate synthetic CT images for the included patients. We hypothesize that the variation in the original training dataset is enough to overcome the post-surgery defects present in our current cohort.

We refer to the original publication for technical details regarding DeepDixon (Ladefoged et al., 2020), but in short the method is a 3D CNN build on the U-Net architecture and trained end-to-end with paired MRI and CT patches consisting of 16 consecutive transaxial slices. The method was implemented in TensorFlow and trained using the Adam optimizer. DeepDixon inference script and trained model weights has been made freely available^1^.

Any region not covered by the CT field-of-view was superimposed by the corresponding area in the atlas-based attenuation map. To ensure a fair comparison, the region was also superimposed on the DeepDixon image.

### 2.6. Image processing and analysis

#### 2.6.1. Delineation of [^18^F]FET-active tumor

The [^18^F]FET-PET images were first normalized to a background region defined in healthy appearing gray and white matter at a level above the insula, automatically extracted by an in-house developed tool designed to match the manual workflow (Ladefoged et al., 2017), see Supplementary material 1. The background region was used to extract individual [^18^F]FET-PET mean values for each of the three AC methods. We further performed skull-stripping on the T1w images using HD-BET (Isensee et al., 2019), and applied the mask to all PET images subsequently to exclude any extracerebral uptake. The BTV of [^18^F]FET-PET was measured using a 3D iso-contour in Mirada XD (Mirada Medical, Oxford, UK) defining tumor tissue at a unique threshold above 1.6 of the mean standardized uptake value (SUV) in the background ROI (Floeth et al., 2005) for each AC method separately. Physiological extratumoral areas with high [^18^F]FET uptake, e.g., vascular structures and pineal body, were identified on either the T1w or PET image and removed from evaluation. Only scans with BTV above 1 ml measured with CT-AC were included in the evaluation of PET accuracy. A total of 222 scans from 147 patients were above the threshold (Table 1).

#### 2.6.2. MR-AC map evaluation metrics

The DeepDixon MR-AC maps (*n* = 318 scans) were manually inspected side-by-side with CT-AC for prevalence of artifacts categorized in four categories: (1) no apparent artifacts, (2) minor not significant artifacts (e.g., small overestimation of titanium clamp), (3) intermediate potentially impactful artifacts (e.g., small metal-induced signal voids), and (4) major artifacts (e.g., large signal voids).

Quantitative performance of DeepDixon and atlas-based AC versus CT-AC were measured by calculating the Dice coefficient for the entire head, limited to the CT field-of-view, as well as areas adjacent to the BTV, defined as a sphere with a maximum distance of 5 cm to the center-of-max of any individual BTV (*n* = 222 scans). For both areas, we calculated the Dice coefficient for both the tissue (0.05–0.1 cm^–1^) and bone (> 0.1 cm^–1^) voxels. Mean absolute error (MAE) and structural similarity index measure (SSIM) was calculated for the entire head.

#### 2.6.3. PET evaluation metrics

The accuracy of the different [^18^F]FET-PET AC methods was assessed on a patient-by-patient basis (*n* = 222 scans) using the guideline recommended semi-quantitative clinical metrics identical to previous studies (Ladefoged et al., 2017, 2019): Mean and maximum SUV and tumor-to-background ratio (TBR) were measured within each BTV, as well as the size of the BTV. These metrics are commonly used as a criterion to identify active tumor tissue from reactive changes (Pöpperl et al., 2006; Langen et al., 2011; Galldiks et al., 2015a,b; Ceccon et al., 2017), and evaluated alongside other factors such as activity and MRI morphology, previous and current treatment, structural changes, and prior imaging results. We adopted the acceptance criteria used in our previous study (Ladefoged et al., 2017): absolute differences of < ± 0.05 and ± 0.1 or relative difference of 5% for mean and maximum TBR, respectively, and ± 2 ml or 10% for the BTV. The mix of both an absolute and relative cut-off reflects that larger absolute difference is acceptable in large or very active tumors.

The size and shape of the BTV has significance for both radiotherapy and surgical planning and the assessment of treatment response (Albert et al., 2016; Moller et al., 2016). Tumor contours relative to the CT-AC reference were analyzed using Dice coefficient and Hausdorff distance metrics, and with a measurement of shape deviations (Supplementary Figure 1), found by thresholding the smoothed tumor difference image:


$$G\,\left(\left|B\,T\,V_{C\,T-A\,C}-B\,T\,V_{X}\right|\right)=1,$$

where *G* is a Gaussian filter with 4 mm FWHM and *X* is atlas or DeepDixon. This is in recognition that the clinical impact of a volume change caused by a focal structure is larger than volume change caused by a one-voxel displacement along the tumor contour.

The location of the TBRmax, which is usually used for biopsy target planning as it identifies the biologically most aggressive component (“hot spots”) in heterogeneous glioma (Messing-Junger et al., 2002; Floeth et al., 2005; Ewelt et al., 2011; Kunz et al., 2011), were compared for each method. Our criterion was set at < 10 mm from the location at our reference PET with CT-AC, as this corresponds to the approximate size of an average stereotactic biopsy.

Finally, global similarity metrics [MAE, SSIM, and peak signal-to-noise ratio (PSNR)] was computed for each MR-AC method against the reference PET with CT-AC within the brain. Relative percent difference was calculated for the whole brain, the BTV, and regionally defined using anatomical pre-defined template regions in Montreal Neurological Institute and Hospital (MNI) (Fonov et al., 2009).

#### 2.6.4. Longitudinal robustness

The robustness of the MR-AC methods over time with importance for response assessment was addressed by calculating the absolute and relative change of TBRmean, TBRmax, and BTV between baseline and follow-up examinations, respectively, and compared to the reference change with CT-AC. A total of 56 patients had at least one follow-up 14 days to 17 months (average: 113 days) following the first. If more than one follow-up was available, we only used the first one. The same clinical acceptance criteria for the longitudinal data as for the single time point data were used.

#### 2.6.5. Dynamic PET imaging

TAC extracted using the 40-min dynamic PET-data were computed for the subset of *n* = 23 scans where this was available. The pattern of the curves, which can be used for diagnosis and treatment response (Dunkl et al., 2015), were visually inspected similarly to previous publications (Galldiks et al., 2013, 2015b), and relative difference to the curves extracted with CT-AC were computed.

### 2.7. Statistical analysis

The correlation between the clinical values (SUV and TBR) extracted using atlas or DeepDixon compared to CT-AC was estimated using the R^2^ coefficient of determination using r2_score function in sklearn (version 1.2.1).

Descriptive statistics of the clinical metrics (TBRmean, TBRmax, and BTV) are provided as mean, 95% confidence intervals (CI), and limits of agreement. The differences were first tested for normality using Shapiro–Wilk test, with *p*-value < 0.05 implying data are significantly different from a normal distribution. Log-transformation was used as data was not distributed normally, and normality was validated using QQ-plots. Exponentiation was applied to the results to express the differences as ratios on the original scale and report them as percentage differences:


$$\text{CI}=100\cdot \left(e^{\frac{d\pm 1.96\,S\,D_{d}}{\sqrt{n}}}-1\right),$$

$$\mathrm{Limits}\,\mathrm{of}\,\mathrm{agreement}=100\cdot \left(e^{d\pm 1.96\,S\,D_{d}}-1\right),$$

where *n* is the number of scans, d is the mean difference, and SD_*d*_ is the standard deviation of the difference on the log scale, where we corrected for repeated measurements from the follow-up examinations (Bland and Altman, 1999).

Paired *t*-test was performed comparing the difference of the log-transformed values of TBRmean, TBRmax, and BTV. A *p*-value < 0.05 indicates statistically significant differences.

All statistical tests were performed using R version 4.2.1.

## 3. Results

**FIGURE 1:**
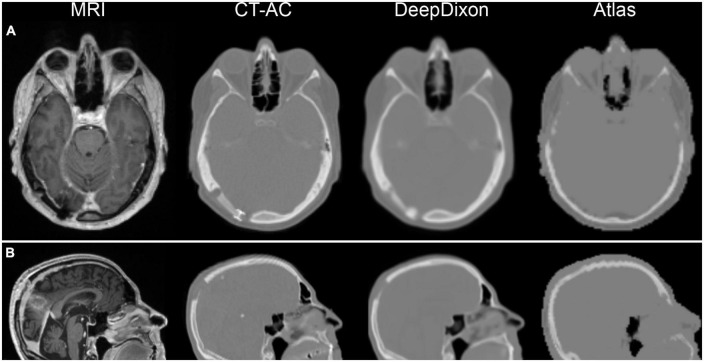
Examples of magnetic resonance imaging (MRI) and corresponding attenuation maps for two post-surgery patients. First patient **(A)** has titanium alloy insert, which shows up has a thickening of tissue on DeepDixon, and not at all in the atlas method. Second patient **(B)** had part of the skull removed in the parietal region, which is well represented by DeepDixon. Particularly the facial and nasal regions are challenged in the atlas method leading to quantitative errors in brain directly bordering the skull base, anterior and posterior fossa, inferior temporal lobes, mesencephalon, pons, and cerebellum.

**FIGURE 2:**
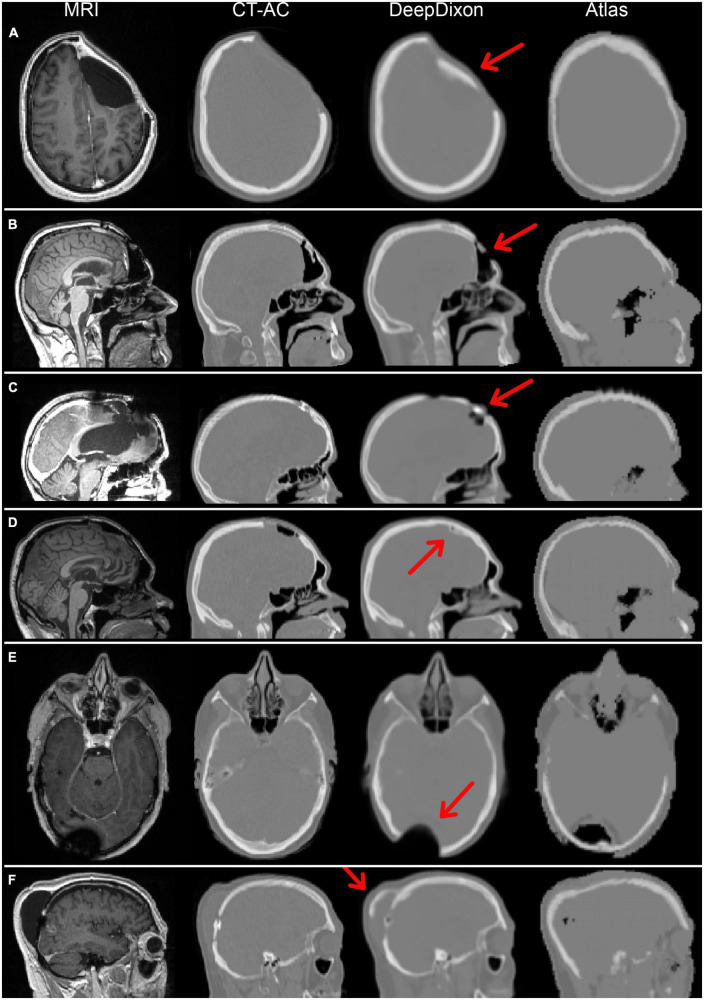
Magnetic resonance imaging (MRI) and attenuation maps for all six patients with DeepDixon artifacts categorized as major. Two patients had follow-up scans, both with consistent artifacts, resulting in a total of 8/318 scans with major artifacts. The patients in **(A–C,E)** had biological tumor volume (BTV) > 1 ml, and were therefore included in the positron emission tomography (PET) evaluation. The primary errors were caused by false bone formation along titanium implant/soft tissue **(A,D,F)**, or directly on brain in postoperative pneumocephalus **(B)**, and metal associated signal voids **(C,E)**.

DeepDixon MR-AC maps were produced for all 318 studies, with an average inference time of 4 s. Visual reading showed that 86% of the studies had no or minor not significant artifacts, with excellent representation of smaller surgical interventions (Figure 1). There were intermediate potentially influential artifacts in 11% of the examinations, most often pronounced as small signal voids at the location of a metal clip, and major artifacts in 3% of the examinations. All images from the major category are shown in Figure 2. The Dice coefficient for DeepDixon was 0.89/0.83 for tissue/bone in the whole head, compared to 0.84/0.57 for atlas. In the vicinity of the BTV, the Dice coefficient was similarly higher for DeepDixon (0.95/0.84) compared to atlas (0.91/0.60) (Supplementary Figure 2).

**FIGURE 3:**
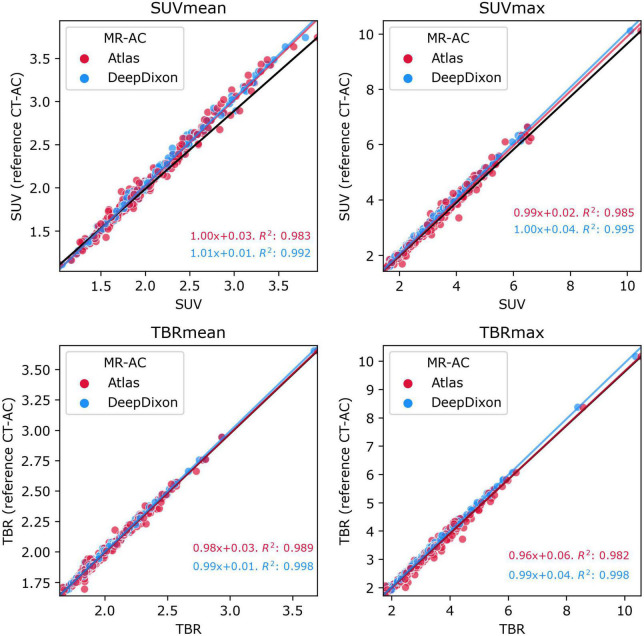
Plot of mean (**left** panels) and max (**right** panels) values within the tumor ROI of [^18^F]FET-PET tissue activity concentration (top panels) and TBR (bottom panels) for atlas and DeepDixon vs. CT-AC reference standard (*n* = 222). The black line indicates the unity line. The goodness-of-determination (R^2^) was calculated including follow-up examinations.

**FIGURE 4:**
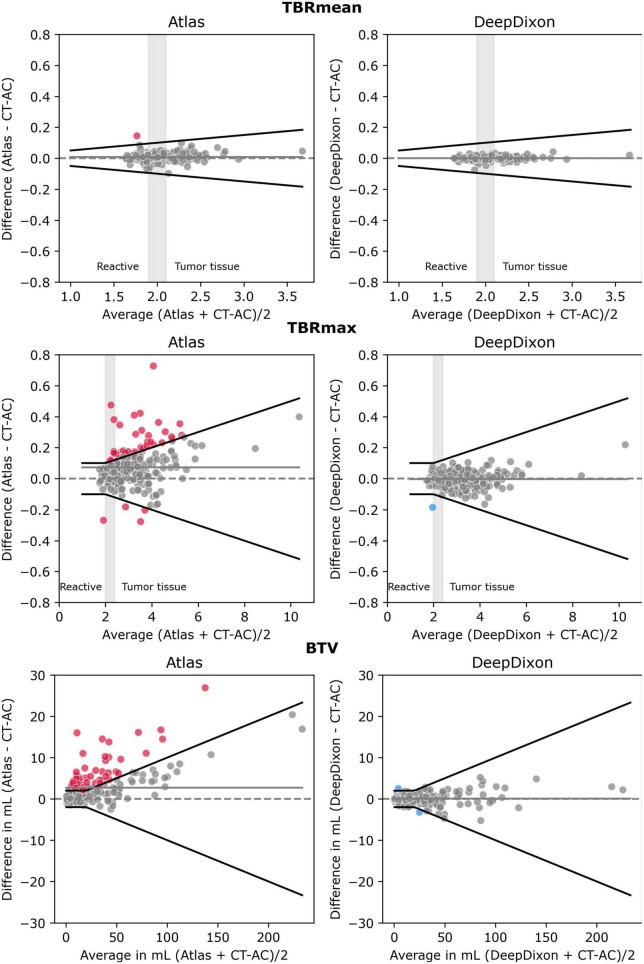
Bland-Altman plot of TBRmean **(top)**, TBRmax **(middle)** and biological tumor volume (BTV) **(bottom)** for each of the two MR-AC methods against the reference standard CT-AC (*n* = 222 scans). To simulate the clinical impact of the metrics in evaluating reactive changes vs. tumor recurrence 3 intervals have been labeled along the *x*-axis for TBRmean and TBRmax. The gray shaded areas define an interval of ambiguity. The black lines indicate the acceptance criteria of TBRmean of ± 0.05 or 5%, TBRmax of ± 0.1 or 5%, or BTV of ± 2 ml or 10%, respectively. Points that exceed the criteria have been colored. The solid gray line indicates the mean value.

The correlation of clinical metrics between the AC methods can be assessed in Figure 3 (*n* = 222). atlas and DeepDixon both recovered the mean and maximum tissue activity concentration as well as TBR within the BTV nearly 100% compared to the reference CT-AC (R^2^ > 0.98 and > 0.99 for atlas and DeepDixon, respectively). Similar R^2^ values were found when only using the baseline examinations (*n* = 147). The difference was statistically significant between atlas and CT-AC but not between DeepDixon and CT-AC (Table 2). The same results were found when the statistical tests were performed on the original data (without log-transformation applied) as well as with only baseline examinations (no follow-up examinations, *n* = 147). When using DeepDixon, only 3 out of 222 studies (1%) exceeded our acceptance criteria of TBRmax difference < ± 0.1 or 5%, TBRmean ± 0.05 or 5%, and BTV ± 2 ml or 10% (Figure 4). These consisted of one patient with TBRmax difference of −0.18 or −9% (2.05 with CT-AC, 1.87 with DeepDixon) caused by metal clips at the location of the lesion and two patients with BTV differences, the worst of which is shown in Figure 2B. When using atlas 72 of the 222 studies (32%) exceeded our acceptance criteria.

**TABLE 2:** Mean relative differences relative to CT-AC of all investigated clinical values (*n* = 222 scans).

Measured parameter values	Mean % difference			95% lower limits of agreement	95% upper limits of agreement
	**Mean**	**95% CI**	***p***		
Atlas					
TBRmean	0.41	0.23 to 0.59	< 0.0001*	−2.19	3.08
TBRmax	2.14	1.63 to 2.66	< 0.0001*	−5.31	10.18
BTV	12.72	9.17 to 16.38	< 0.0001*	−30	81.51
**DeepDixon**					
TBRmean	−0.02	−0.10 to 0.06	0.64	−1.27	1.24
TBRmax	−0.19	−0.41 to 0.02	0.08	−3.38	3.09
BTV	−0.11	−1.58 to 1.38	0.88	−19.9	24.56

Exponentiation was applied to the results from analyses on log scale and expressed as percentages. *Indicates a statistical significance (P < 0.05) from 0 found by a paired t-test. The paired t-test was performed included follow-up examinations. CI = 95% confidence interval for mean difference.

When using TBR to differentiate between reactive changes and tumor tissue, TBRmax < 2.0 is often considered reactive tissue whereas TBRmax > 2.4 and TBRmean ∼ > 2.0 is considered indicative of active tumor tissue. When applying these thresholds atlas, DeepDixon, and CT-AC had concordant classifications (reactive vs. tumor tissue) in all studies. Minor differences resulted in an equivocal classification (TBRmean between 1.9 and 2.1, TBRmax between 2 and 2.4) being changed to reactive or tumor tissue, or vice versa in 26 studies using atlas and 10 studies using DeepDixon. The absolute difference value range for these 26 patients using atlas to have the same category as the reference was 0–0.21 for TBRmax (*n* = 10) and 0.02–0.05 for TBRmean (*n* = 16). In comparison, the same range for DeepDixon was 0.03–0.13 for TBRmax (*n* = 5) and 0.01–0.06 for TBRmean (*n* = 5).

The tumor delineation precision was improved, from Dice coefficient of 0.90 ± 0.1 and Hausdorff distance 2.7 ± 3.5 mm using atlas, to 0.95 ± 0.1 and 1.3 ± 2.9 mm, respectively, using DeepDixon (Supplementary Figure 3). The shape deviation analysis found that only one study had distinct warps in the outline of the BTV of more than 1 ml (1.5 ml) using atlas and none using DeepDixon. The peak location of TBRmax used for biopsy guidance was in general in agreement; 91% were within 10 mm compared to CT-AC using atlas and 94% using DeepDixon. While the peak location for the remaining studies was between 10 and 115 mm from the reference, TBRmax was nearly identical in all studies when comparing reference and identified peak location, with a maximum relative difference of 4.4% for atlas (*n* = 25) and 3.5% for DeepDixon (*n* = 7).

The percent change between baseline and follow-up examinations is shown for each of the AC methods in Supplementary Table 1 for the 56 patients with at least one follow-up examination. TBRmean was congruent in all patients using either MR-AC method. For TBRmax, the difference in percent change was within our 5%-point acceptance limit for all patients with DeepDixon, but was exceeded in 8 patients using atlas (range: 5–24% or 0.1–0.7 in absolute values), however, all with the same change direction as CT-AC. The absolute percentage point difference exceeded our acceptance limit of 10%-point and 2 ml for BTV in 3 patients with DeepDixon (range: 12–42% or 2–3 ml) and 10 patients with atlas (range: 10–260% or 2–7 ml). The direction of the volume change was congruent with CT-AC when using DeepDixon, while atlas resulted in discordant direction in 5 patients, the worst being an increase of 0.6 ml (5–5.6 ml) when measured with CT-AC whereas using atlas resulted in a 3 ml decrease (9–6 ml).

**FIGURE 5:**
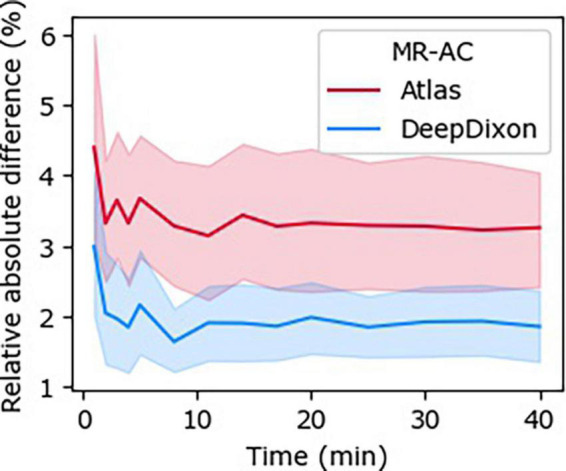
Relative absolute difference between time-activity curves (TACs) across all subjects (*n* = 23) to the dynamic 40-min positron emission tomography (PET) with CT-AC reference for each of DeepDixon and atlas MR-AC, respectively.

The relative absolute difference for the 23 patients with 40-min dynamic PET data was 2.0 ± 1.6% from CT-AC across all patients and time-points when using DeepDixon compared to 3.4 ± 2.4% when using atlas (Figure 5), with the largest bias for a single patient and time-point of 10% for DeepDixon and 17% for atlas. All TAC for both methods followed a course parallel with CT-AC. Thus, there were no change of TAC configuration.

Globally, DeepDixon has an improved PSNR, SSIM, and MAE over atlas compared to CT-AC on both the attenuation map and resulting PET images (Supplementary Table 2). Global and regional relative difference evaluation is shown in Supplementary Figure 4.

## 4. Discussion

This study evaluated the accuracy of our deep learning-based MR-AC method for [^18^F]FET-PET in a large group of post-surgery glioma patients. This category of patients is a challenge to MR-AC methods due to the presence of gross anatomical deformations and metal implant-induced susceptibility artifacts. At the same time the use of well-established and recommended (semi-) quantitative metrics makes these patients ideal for a clinical evaluation.

Overall, we found that DeepDixon, despite being trained on data without irregular anatomy or metal artifacts, robustly reproduced the clinical metrics found with CT-AC. Irregular anatomy and titanium alloy clamps were accurately represented by DeepDixon in most patients, with only minor deviations that did not impact the surrounding PET tracer uptake. The vendor-provided atlas MR-AC method achieved acceptable performance on average, but patient-by-patient evaluation revealed significant outliers that might compromise accurate diagnosis in border-line cases.

Visual inspection of attenuation maps for artifacts, consistency and plausibility is always recommended in PET/MRI (Law et al., 2019). We found eight scans from six patients with major artifacts in DeepDixon that might change the tracer uptake and subsequently the clinical metrics, of which four scans had BTV > 1 ml and were therefore included in the PET evaluation (Figure 2). All metrics were within the acceptable limits in three of these patients despite the artifacts, in part due to the distance between artifact and BTV being 2, 7, and 10 cm. The final patient (Figure 2B) had an increase in BTV from 2.9 ml with CT-AC to 5.4 ml with DeepDixon due to the appearance of a 1–2 voxel elongated region above threshold in the frontal cortex (Supplementary Figure 5). This patient had frontal postsurgical pneumocephalus that was likely to have been interpreted by the AI-method as the frontal sinuses causing erroneous bone formation to be built. The same patient had a significantly larger error using atlas MR-AC with a BTV of 18.9 ml, as the air space was filled by the attenuation value of water. While visual inspection of the MR-AC maps remains of importance, considering the distance between the tumor and artifact can be helpful in estimating the introduced bias. Using atlas MR-AC metrics exceeded the acceptable limits in three of the four patients. Overall, the prevalence of patients with metrics that exceeded our acceptance criteria using DeepDixon (*n* = 3, 1%) is even lower than our previous findings using RESOLUTE for MR-AC, where 5 out of 68 (7%) exceeded the acceptance criteria (Ladefoged et al., 2017).

The results of the follow-up analysis showed that both MR-AC methods robustly reproduced the magnitude and direction of the change between scans, albeit with DeepDixon being the most accurate, thus leading to similar conclusion of treatment response regardless of AC method applied. This indicates that DeepDixon can replace CT-AC even in neuro oncological response assessment. In a separate analysis (results not shown) we evaluated the impact of changing to DeepDixon in the follow-up examination when using CT-AC for the baseline examination and found results that were similar to using DeepDixon for both baseline and follow-up. Low between scanner variability is important for the method reliability and practical use as [^18^F]FET PET/MRI and PET/CT will often be used interchangeably clinically for response assessment.

On average, the vendor-provided atlas-based MR-AC method produced clinical metrics that are comparable to the CT-AC reference, with TBRmean and TBRmax relative differences of 1–3%. These results confirm the findings of Rausch et al. (2017) in 24 patients, that found a relative difference of 0 ± 2 and 0 ± 5% between atlas MR-AC and CT-AC for TBRmean and TBRmax, respectively. When evaluated on a patient-by-patient basis, however, almost a third of our scans had clinical metrics outside the acceptable limits, which underlines the importance of evaluation on a single subject level and in a large patient group to embrace the variation in artifacts. The errors were most often caused by metal implant-induced signal voids and irregular anatomy challenging the registration accuracy, also supported by the poor Dice score for bone in areas near the BTV often affected by surgical intervention (Supplementary Figure 2).

In 6–9% of patients the peak location deviated more than 10 mm, which would impact biopsy planning. These were larger (BTV > 10 ml) and irregular tumors often close to the resection cavity, where the peak could “slide” along a ridge. The TBRmax did not change, but we have no way of assessing the consequences in underlying sampled histology. It should be noted that surgical biopsies are mostly performed under more ideal conditions in preoperative patients with more homogenous intact tumors without surgical fragmentation, postoperative treatment related changes and cranial modifications, where precision may be even higher. Postsurgical biopsies will usually not be directed toward recurrence in a resection cavity, but toward distinct, deeper seated and smaller localized suspected recurrences. Thus, although our patient group is not representative of the typical biopsy candidates, they do give an indication of performance under difficult conditions.

The performance of the atlas MR-AC method for dynamic data was also revaluated by Rausch et al. (2019). Here, the authors categorized the TACs into three categories (increasing, plateau, and decreasing) depending on the shape of the curve. The authors found a change in the TAC pattern, compared to a CT-AC reference, in one of the 17 cases they evaluated. We did not find any difference between the TAC shapes regardless of AC method in the BTV or the reference region. Direct comparison between that study and ours is challenging, due to differences in BTV delineation and number of frames, but overall our evaluation confirmed the previous findings in that the TACs are robust toward choice of MR-AC method using either atlas or DeepDixon, which is important for both diagnosis (Galldiks et al., 2015b; Ceccon et al., 2017) and prognostic prediction (Bauer et al., 2020).

We have previously evaluated our deep learning method on a post-surgery pediatric brain tumor cohort (Ladefoged et al., 2019), and concluded that it could robustly represent even irregular anatomy present in the dataset, resulting in clinical metrics on par with CT-AC. The pediatric model was trained using fourfold cross validation on 79 scans, including cases with severe abnormal anatomy. Since our dataset included cases with major artifacts (Figure 2), we attempted to apply transfer learning of the adult DeepDixon model to our current oncology dataset using fourfold cross-validation, where each hold-out fold contained 2 scans with major DeepDixon artifacts identified. We did not see any real improvement (results not shown), which likely has to do with the low prevalence of the gross artifacts.

In clinical practice a pragmatic and cost-effective strategy can be employed similar to the one we presently use in PET/MRI of our dementia patients (Ladefoged et al., 2020). The fast generation of a DeepDixon MR-AC map allows early artifact screening by technologist trained on the examples in this paper and the subsequent acquisition of low-dose CT for CT-AC in relevant patients without compromising clinical quality compared to PET/CT. We have in our unit used DeepDixon for primary brain MR-AC of [^18^F]FDG-PET/MR evaluations of dementia patients for more than 3 years generating over 1,500 attenuation maps. The results of this study substantiate that DeepDixon is ready for routine clinical implementation for MR-AC even under more challenging conditions such as post-surgical glioma patients.

### 4.1. Limitations

Our patient cohort predominantly consisted of [^18^F]FET-PET scans with clear indications of viable tumor tissue of which 114 (50%) had TBRmax ratio above 3.0. For these patients, large deviations do not lead to a change in clinical reading. Only 23 scans had data acquired for the full 40-min uptake period. More patients should be included to determine the impact of AC on the dynamic biomarkers. Our study does not compare the performance of DeepDixon against other atlas- or segmentation-based state-of-the-art methods.

## 5. Conclusion

This study is the largest study to date evaluating the performance of a state-of-the-art MR-AC method on post-surgical glioma patients scanned using simultaneously acquired [^18^F]FET-PET/MRI. We found that DeepDixon could overcome most of the issues arising from irregular anatomy and metal artifacts present in the cohort, resulting in clinical metrics within acceptable limits of the reference CT-AC in almost all cases, which is an improvement over the vendor-provided atlas method. Using follow-up scans we found DeepDixon to be robust in neuro oncology response assessment.

## Data availability statement

The datasets presented in this article are not readily available because the data contain patient identifiable information. Requests to access the datasets should be directed to IL, ian.law@regionh.dk.

## Ethics statement

Ethical review and approval was not required for the study on human participants in accordance with the local legislation and institutional requirements. Written informed consent for participation was not required for this study in accordance with the national legislation and the institutional requirements.

## Author contributions

CL designed the method, did the data analysis, and prepared the manuscript. LA and CE aided in the data analysis, revised, and approved the manuscript. FA, TA, KM, LH, OH, and IL aided in the data acquisition, data analysis, revised, and approved the manuscript. All authors contributed to the article and approved the submitted version.

## Footnotes

1
https://github.com/CAAI/DeepMRAC
