# Supplementary material for: DeepDixon synthetic CT for [18F]FET PET/MRI attenuation correction of post-surgery glioma patients with metal implants
Source: Front Neurosci. 2023 Apr 6;17:1142383. doi: 10.3389/fnins.2023.1142383 (PMC10115992; doi:10.3389/fnins.2023.1142383)
Supplement: Supplementary file 1 [file Data_Sheet_1.docx]

Supplementary Material

DeepDixon synthetic CT for [^18^F]FET PET/MRI attenuation correction of post-surgery glioma patients with metal implants

Claes Nøhr Ladefoged^*^, Flemming Littrup Andersen, Thomas Lund Andersen, Lasse Anderberg, Christian Engkebølle, Karine Madsen, Liselotte Højgaard, Otto Henriksen, Ian Law

*** Correspondence:** Claes Nøhr Ladefoged. claes.noehr.ladefoged@regionh.dk

Supplementary material

# S1: Generation of background region of interest used for TBR calculation

The procedure first aligns the PET and T1w images to MNI space, in which four candidate regions were predefined in the left and right anterior and posterior regions. Next, the PET image is used to select the two regions with the lowest mean uptake if the value of these were similar, or the single region with the lowest activity otherwise. Manual inspections of the regions are performed to ensure that the background region is only placed in healthy tissue. An example of the report generated for each case used for inspection is shown below.


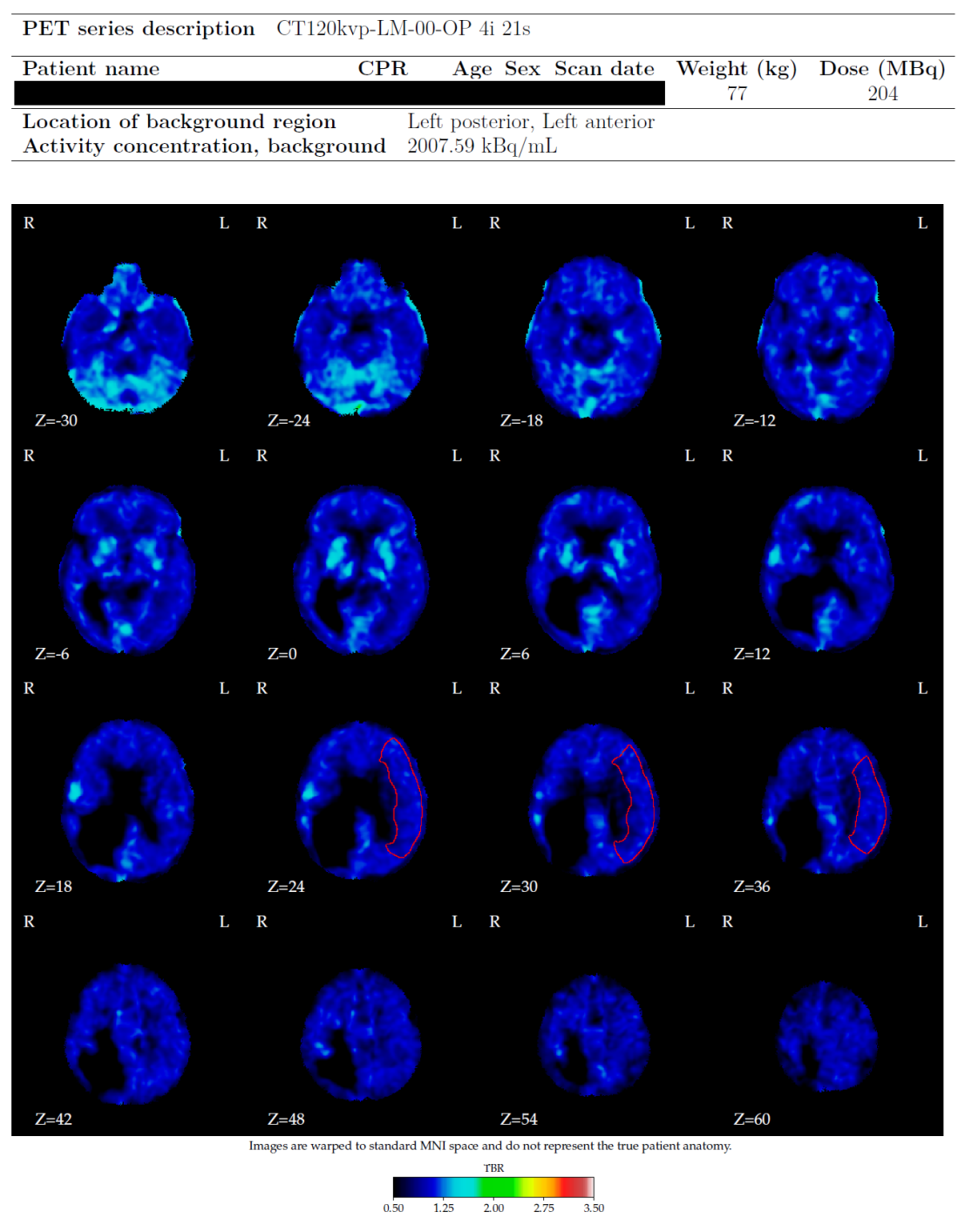


# Supplementary Tables

**Supplementary Table 2**: Image similarity evaluation on attenuation map and PET images for each MR-AC method compared to CT-AC reference. Values in table indicate mean ± standard deviation.

|  | **Attenuation map metrics** | | **PET image metrics** | | |
| --- | --- | --- | --- | --- | --- |
| **MR-AC** | **MAE** | **SSIM** | **MAE** | **SSIM** | **PSNR** |
| Atlas | 150 ± 25 | 0.873 ± 0.018 | 116 ± 34 | 0.996 ± 0.001 | 44 ± 3 |
| DeepDixon | 84 ± 32 | 0.928 ± 0.022 | 60 ± 35 | 0.999 ± 0.001 | 51 ± 4 |

MAE: Mean absolute error
SSIM: Structural Similarity Index Measure
PSNR: Peak signal-to-noise ratio

# Supplementary Figures

| 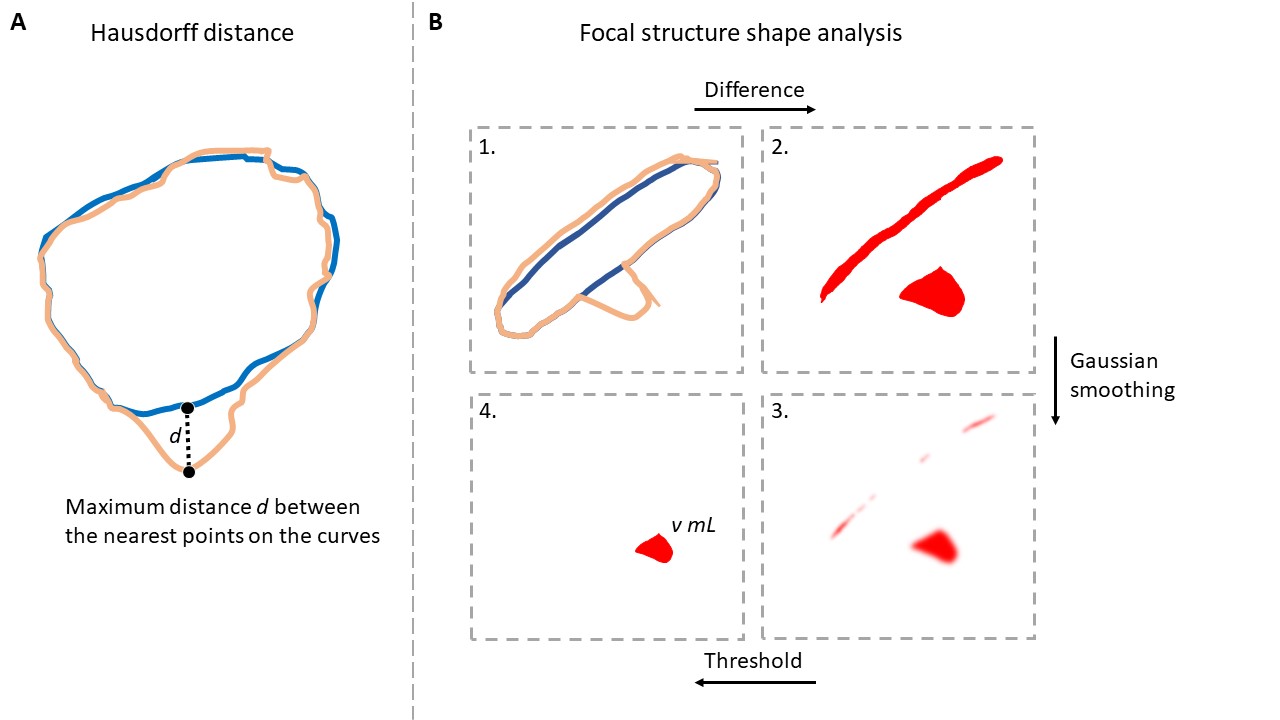 |
| --- |
| **Supplementary Figure 1**: Illustration of shape deviation analysis. Left panel (A) shows the Hausdorff distance defined by the maximum distance *d* between the nearest points on two curves. Right panel (B) shows the analysis of shape deviation robust to gaussian blurring. First the shapes (1.) is subtracted to find the difference (2.) followed by a 4 mm Gaussian smoothing (3.) and a thresholding of values equal to 1 (4.). The remaining volume represent a larger focal structure which is measured in mL. |

| 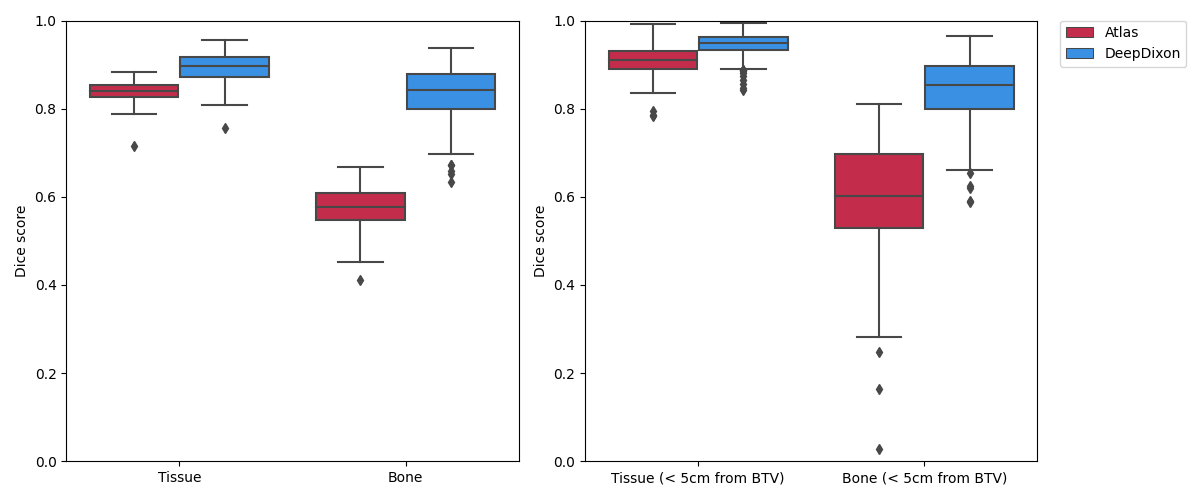 |
| --- |
| **Supplementary Figure 2**: Dice for MR-AC maps in CT-FOV (left) and within 5 cm from CoM of each BTV for the 222 studies with BTV > 1 mL. |

| 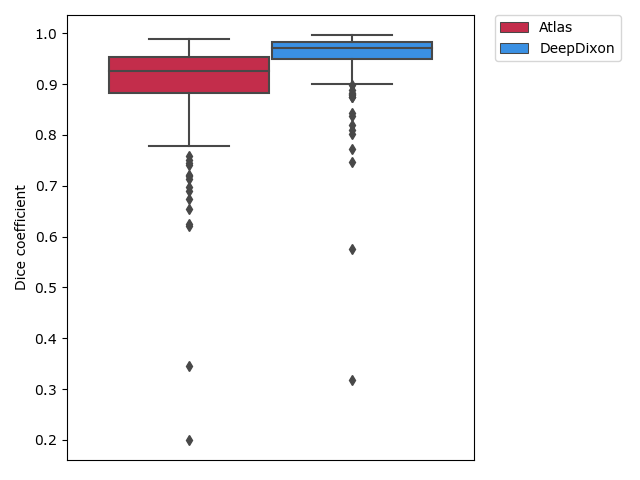 | 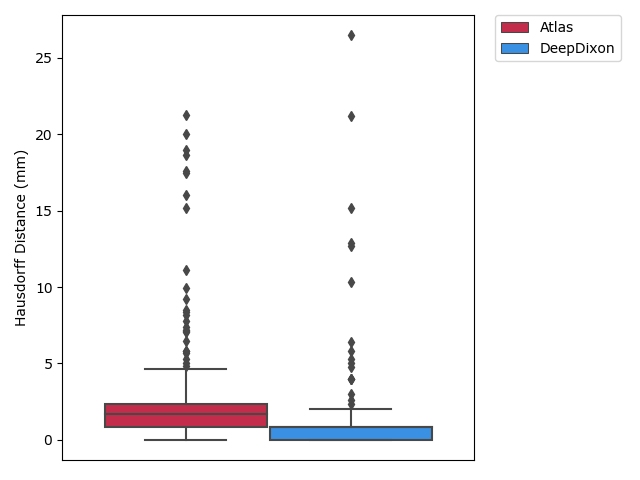 |
| --- | --- |
| **Supplementary Figure 3**: Similarity metrics Dice coefficient and Hausdorff distance for BTV compared to reference CT-AC for the 222 studies with BTV > 1 mL.  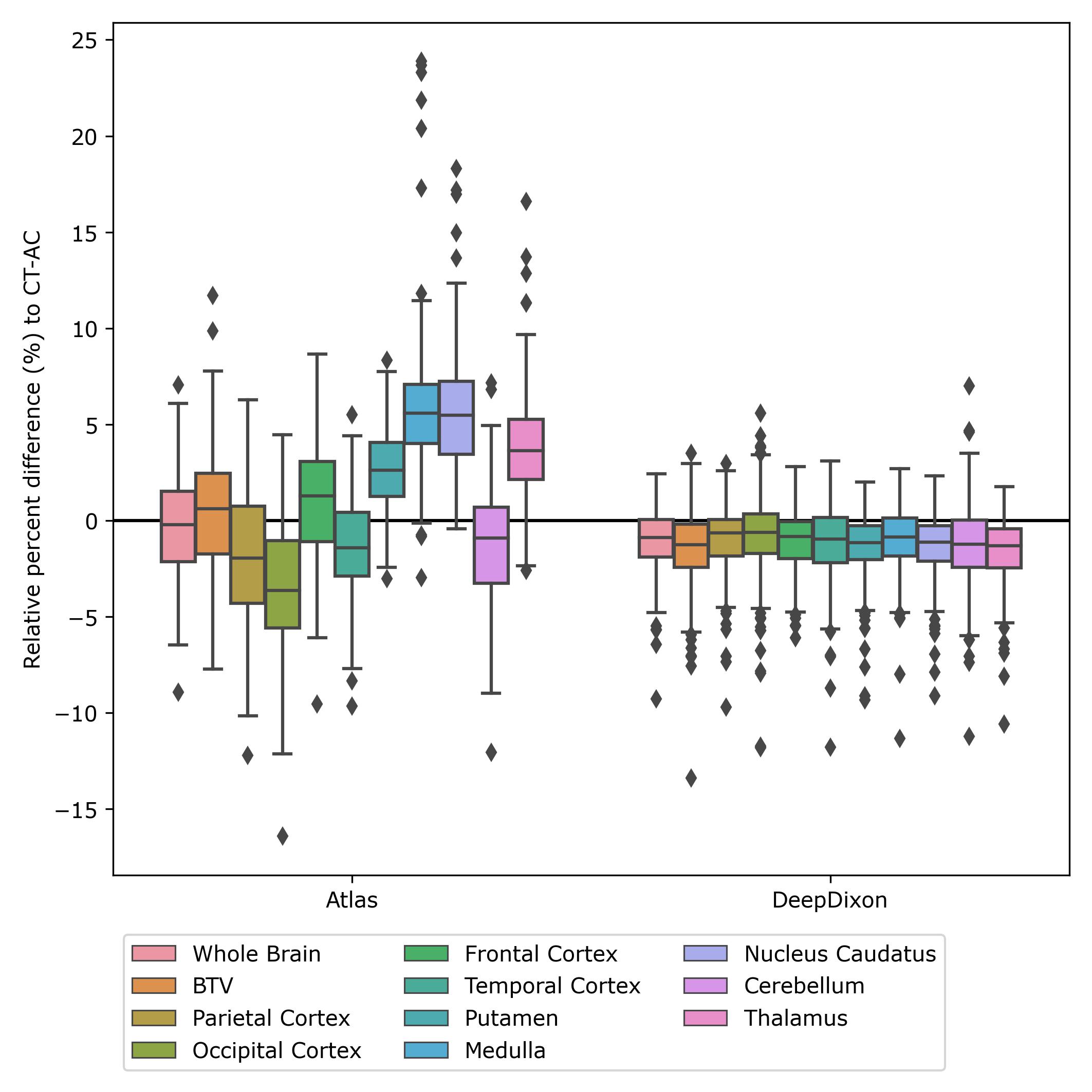 | |

**Supplementary Figure 4:** Whole brain and regional mean relative differences across all patients with BTV > 1 mL (n=222) for each of the two MR-AC methods Atlas and DeepDixon compared to CT-AC reference.

| 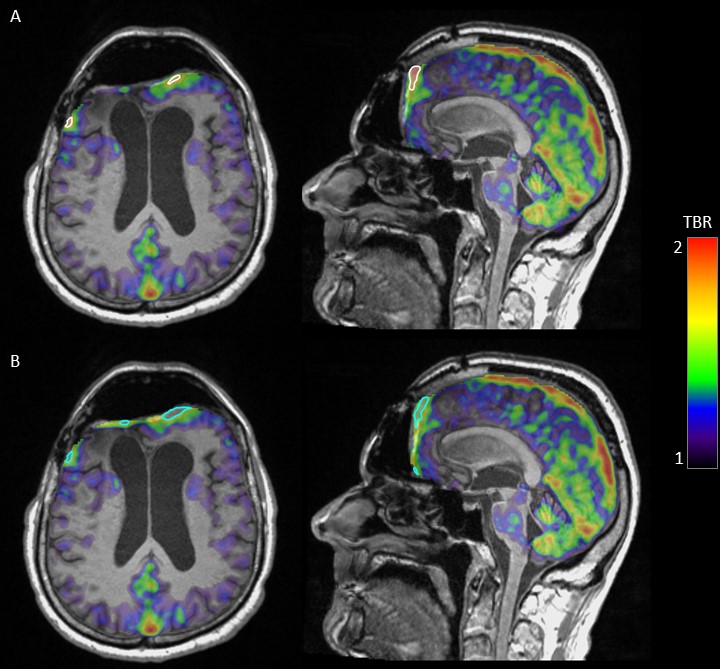 |
| --- |
| **Supplementary Figure 5**: [^18^F]FET PET/MRI of patient with large artifact corresponding to Fig. 2 B. The top panels shows the PET image with CT-AC overlaid onto the T1w MPRAGE and the bottom panels show the PET image with DeepDixon for MR-AC. BTV is larger when using DeepDixon (5 mL, cyan on bottom panel) compared to CT-AC (3 mL, white on top panel). The patient had postsurgical pneumocephalus leading to the formation of a false bone layer on the frontal cortex. |
